# Ketogenic diet enhances the effects of oxycodone in mice

**DOI:** 10.1038/s41598-023-33458-8

**Published:** 2023-05-09

**Authors:** R. Trinko, D. M. Diaz, E. Foscue, S. L. Thompson, J. R. Taylor, R. J. DiLeone

**Affiliations:** Department of Psychiatry, Yale University School of Medicine, New Haven, CT 06519 USA

**Keywords:** Reward, Addiction

## Abstract

Opioids have been used to manage pain for thousands of years, but they have significant potential for abuse. Prescription opioids, like oxycodone, are associated with 32% of overdoses, that have reached a total of 75,673 deaths in 2021. A major challenge is maximizing their therapeutic potential while minimizing the negative side effects including opioid use disorder (OUD). The Ketogenic Diet (KD) has been reported to reduce pain and decrease the severity of alcohol use disorder, yet its effects on oxycodone responses remain unknown. KD mice displayed increased oxycodone-induced locomotor activity and enhanced antinociceptive effects of oxycodone, suggesting a dietary effect on opiate sensitivity. Male KD mice exposed to chronic oxycodone exhibited increased naloxone-induced jumps, suggesting a sex-specific effect of diet on opioid withdrawal. Consistent with this, male KD mice self-administered less oxycodone while female KD mice did not differ from controls. Finally, no effect of KD on motivation to obtain oxycodone was observed during a progressive ratio schedule. These data suggest sex-biased effects of KD on responses to opioids that should be considered and potentially leveraged in both clinical pain management and treatment of OUD.

## Introduction

The prevalence of opioid overdose deaths continues to rise dramatically, making it a major public health concern^1^. Opioid overdose deaths increased 519% between 1999 and 2019, with 56,064 deaths reported in 2020 and 75,673 in 2021^2,3^. Prescription opioids such as oxycodone have played a major role in the opioid epidemic, contributing to 32% of overdose deaths^3^. In the United States more than 2 million individuals have a substance use disorder related to prescription opioids; of patients prescribed opioids, 25% misuse them, and 5–10% develop OUD^4^. Despite the high risk of abuse associated with oxycodone, it is still one of the most effective and prescribed medications for pain management^5^. In reaction to the opioid crisis, greater attention has been placed on investigating effective treatments for OUD and maximizing the therapeutic benefit of oxycodone while minimizing the negative consequences in a pain management setting.

Medication-assisted treatment (MAT) for OUD currently utilizes three FDA-approved medications: (1) methadone, (2) buprenorphine, (3) and extended-release naltrexone^6^. Methadone and buprenorphine are opioid agonists at the mu-opioid receptor, reduce opioid use, craving, and mortality, while increasing treatment retention^6^. However, both drugs are prone to abuse by patients^7^. Naltrexone, an opioid antagonist, is available in oral and extended-release injectable forms (Vivitrol)^6^. Vivitrol treatment results in reduced opioid craving and use, but does not reduce the risk of mortality, and unfortunately has the lowest treatment adherence likely due to a required withdrawal period prior to initiation^6^. Given the public health crisis, it is critical to both develop more effective therapeutics, and to better understand how physiological states modify OUD and response to therapeutics.

It is well established that nutritional deficiencies stemming from poor diet are highly prevalent in the OUD population and may contribute to impaired recovery^8–13^. Incorporating a nutritional component to OUD treatment may improve patient outcomes and decrease healthcare costs^11^. However, nutritional interventions for substance use disorder (SUD) are still in their infancy, as very few studies have investigated dietary manipulation as a potential treatment adjunct for SUD. Consequently, it is difficult for healthcare providers to give specific and effective nutritional recommendations. The Ketogenic Diet (KD) is a high fat, low/moderate protein, low carbohydrate diet, best known for its efficacy in treating pediatric epilepsy^14^. KD has recently been demonstrated to have potential for improving alcohol withdrawal symptoms in clinical^15^ and preclinical^16,17^ studies, and was found to reduce alcohol self-administration in rodents^15,18,19^. Notably, there is also preliminary evidence that KD, outside of opiates, has antinociceptive benefits in preclinical^20,21^, as well clinical settings^22^. Together, this evidence suggests the possibility that KD could modulate the oxycodone response. Yet, the role of KD in effects of oxycodone is unknown. Here, we sought to identify the potential effects of a KD on a range of oxycodone responses in mice.

## Methods

### Animals and diets

Animal experiments were conducted in accordance with the Yale University School of Medicine Institutional Animal Care and Use Committee (IACUC) animal care guidelines. All protocols were approved by Yale IACUC. All methods are reported in accordance with ARRIVE guidelines. No animals were removed from analysis. The experimenters were not blinded to the dietary groups. Male and female C57BL/6 J mice, purchased from The Jackson Laboratory, were between the ages of 8 and 15 weeks for the experiments. Separate cohorts of animals were used for self-administration and osmotic minipump studies. Mice were group housed up to five per cage on a 12 h light/dark cycle (lights on at 7:00 A.M.).

Ketogenic Diet AIN-76A Modified (Bio-Serv #F3666) had a caloric value of 7.24 kcal/g, with a composition of 93.4% fat, 4.7% protein, and 1.8% carbs. Standard chow (Teklad Global #2018) had a caloric value of 3.1 kcal/g, with a composition of 18% fat, 24% protein, and 58% carbs. All mice were maintained either ad libitum on these diets, restricted on Chow to accomplish matching bodyweight changes (pair-fed mice), or restricted to maintain 85–90% of starting bodyweight for self-administration studies. For the studies involving paired-feeding, male pair-fed mice were food restricted on standard chow to match the percent change in bodyweight loss observed in the KD males prior to experimentation, which we observed to be close to 90%. This matching was maintained daily throughout the course of dietary intervention, locomotor activity, and then hot plate testing.

### Statistics

GraphPad Prism 9.0 and RStudio v1.4.1717-3^23^ were used to perform statistical tests. For *t-*tests, datasets were first assessed for normal Gaussian distribution. Mann–Whitney U analysis was conducted for datasets that were not distributed normally. For most binned tests with repeated measures, a repeated measures two-way ANOVA was used, with Sidak’s and Tukey’s post hoc analysis conducted when applicable. Specifically, Tukey’s was used for the analysis of more than two groups. Because the hot plate experiments utilized a maximum 40 s cutoff for latency, a Cox mixed-effects proportional hazards model with timepoints set as numeric was used for the analysis of data points after oxycodone injection, with R packages coxme and multcomp^24,25^. Body weight change analysis for males that included a Pair-fed group was assessed using a one-way ANOVA.

### Ketone levels

Tail blood was used to assess ketone levels using a Precision Xtra ketone meter (model #P0BF9) or a Keto Mojo ketone meter (model # 21335C002AEEB) and used according to the manufacturer’s specifications.

### Drugs

Saccharin (Acros Organics #149005000) was diluted to several concentrations (the highest being 0.1%) in tap water. Oxycodone HCl was supplied by the NIDA drug sharing program. Oxycodone was diluted at various concentrations in either tap water for self-administration experiments, or sterile saline for injections and osmotic minipump loading. Naloxone Hydrochloride was purchased from Spectrum Chemical (#N1231), dissolved in sterile saline, and injected at 1 mg/kg intraperitoneal (i.p.) immediately prior to assessing jumping behavior. Lidocaine (Covetrus, North America VINB-0024-6800) was injected locally at 4 mg/kg subcutaneously (s.c.) prior to incision for minipump implantation. Carprofen (Zoetis) was injected i.p. at 5 mg/kg prior to incision for minipump implantation, and again at 24 and 48 h after surgery.

### Locomotor activity

Med Associates locomotor chambers, which utilize infrared beams, and Med PC V software were used to assess locomotor activity. All mice were habituated to Med Associates locomotor chambers for 90 min, during which they all received a saline injection i.p. at 60 min. Following the 90 min habituation period, mice then received an acute 2 mg/kg oxycodone injection i.p., and activity was measured over an additional 90 min in 5 min bins. Two consecutive beam breaks were required to register as locomotor activity.

### Hot plate assay

Mice that were previously used for locomotor activity were assessed for nociception, with a minimum of two days between tests. The hot plate assay was conducted using a Series 8 hot plate from IITC Life Science held at a constant 52 °C. A 40 s cutoff time was implemented to avoid tissue damage. Nociceptive measures included paw flick, paw lick, or jumps. Each mouse was immediately removed from the hot plate after the first incidence of nociception or 40 s, whichever came first. Baseline nociception was tested first. Then 2 mg/kg oxycodone was delivered i.p. and nociception was tested at 10, 20, 30, and 45 min post-oxycodone.

### Precipitated withdrawal

Osmotic minipumps (Alzet #1003D) were used to chronically deliver oxycodone over three days. Mice were placed on their respective diets ad libitum for four days prior to osmotic minipump surgeries. The day before surgery, bodyweights were assessed, and minipumps were custom loaded to deliver 130 mg/kg/day. Ketone levels were measured prior to surgery. Surgeries were performed under aseptic conditions. Briefly, mice were injected with carprofen, anesthetized with isoflurane, and shaved at the incision site on their backside lateral from their spine. The exposed skin was cleaned with betadine, lidocaine was injected locally, and a small incision was made with scissors. A subcutaneous pocket was made and the minipump was inserted. Wound clips were used to close the incision site and triple antibiotic cream was applied. Animal welfare was monitored daily following surgery. Three days after surgery, mice were injected with 1 mg/kg naloxone i.p. and placed in an empty static mouse bucket with a high lid. Jumping behavior was chosen as our measure of withdrawal as this has consistently been the most objective and quantifiable outcome observed in our lab. Jumps were quantified using Med Associates locomotor chambers, with the beam detectors raised approximately six inches off the ground, high enough to require all four paws leaving the cage floor, and to not detect rearing. A jump was counted when an animal broke a beam, with a 0.5 s timeout period to allow for the mouse to land. Jumps were quantified over 20 min.

### Oxycodone self-administration and progressive ratio

All mice were maintained on standard chow and food restricted to 85–90% of their bodyweight. Med Associates operant boxes were used, which were equipped with three nosepoke ports on one side, a magazine for reward retrieval on the opposite wall, cue lights in the active nosepoke port and magazine, and a syringe pump for delivery of liquid rewards. Five ml syringes were used, and pump activation time was set to deliver 20 µl of liquid into the magazine per reward earned. The operant sequence of events started with activation of the nosepoke port as indicated by illumination of its cue light. Upon a successful nosepoke port entry, the nosepoke cue light was turned off, a reward was immediately delivered and a cue light in the magazine was turned on. Upon successful magazine entry by the mouse, the magazine cue light was turned off, and after a 5 s delay, the appropriate nosepoke port was made active again, as indicated by its cue light. Self-administration sessions were 3 h long. Mice were initially trained to self-administer 0.1% saccharin solution in water on a fixed ratio 1 (FR1) schedule, in which each active nosepoke resulted in the delivery of saccharin reward, which then was increased to an FR3 schedule (3 nosepokes per reward). After several days of FR3 0.1% saccharin, oxycodone was gradually introduced over several days, from 0.05 mg/ml to 0.3 mg/ml. After four days at 0.1% saccharin 0.3 mg/ml oxycodone, the saccharin was fully faded out over four days. After six additional days of 3 h sessions at FR3 with unsweetened oxycodone, mice were assigned to their dietary groups (KD or Chow), counterbalanced based on their oxycodone consumption. Self-administration sessions and food restriction were maintained throughout the dietary transition. Magazines were checked at the end of each session to verify rewards had been consumed. Mice were then tested on an exponentially increasing progressive ratio schedule^26–28^. The session was conducted over 6 h, with drinking water provided in the operant box to eliminate thirst as a confound for liquid seeking. No cutoff time was implemented in the program. The nosepoke requirement schedule (where j = 0.2) for the first 14 rewards is as follows: 1, 2, 4, 6, 9, 12, 15, 20, 25, 32, 40, 50, 62, 77.

## Results

### Progression of ketosis and the effects of KD on bodyweight

**Figure 1:**
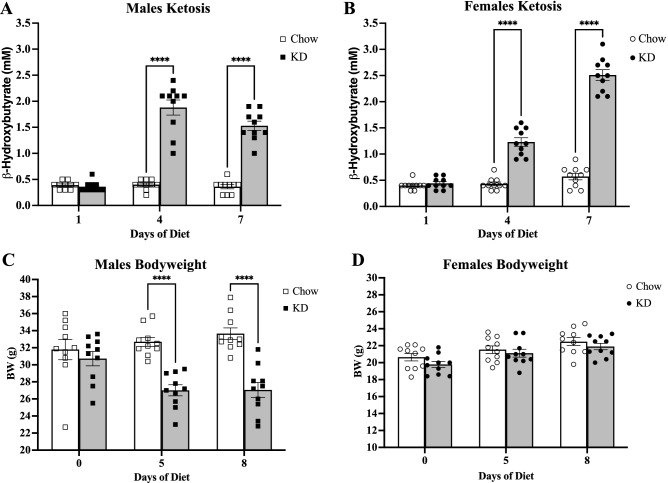
Typical progression of ketosis after diet initiation, and sex difference in the effects of KD on bodyweight. (**A**, **B**) β-hydroxybutyrate levels from tail blood for males (n = 10, 10) and females (n = 10, 10) over time. A two-way repeated measures ANOVA revealed a main effect of diet (males, *F*(1, 18) = 160.6, *p* < .0001; females, *F*(1, 18) = 410.1, *p* < 0.0001) and time (males, *F*(1.668, 30.02) = 67.24, *p* < 0.0001; females, *F*(1.243, 22.38) = 143.5, *p* < 0.0001) with an interaction of diet x time (males, *F*(2, 36) = 67.66, *p* < .0001; females, *F*(2, 36) = 102.2, *p* < 0.0001) for ketone levels. A Sidak’s multiple comparisons test revealed a highly significant increase in ketones on day 4 (males ****p < 0.0001; females ****p < 0.0001) and day 7 (males ****p < 0.0001; females ****p < 0.0001). (**C**) Bodyweight changes in male mice (n = 10, 10) over time. A two-way repeated measures ANOVA revealed a main effect of diet (*F*(1, 18) = 18.98, *p* = 0.0004) and time (*F*(1.266, 22.78) = 4.386, *p* = 0.0395) with an interaction of diet x time (*F*(2, 36) = 19.13, *p* < 0.0001). A Sidak’s post hoc analysis showed significant bodyweight differences at Days 5 and 8 post-diet induction (****p < 0.0001). (**D**) Bodyweight changes in female mice (n = 10,10) over time. A two-way ANOVA analysis for sex differences of net bodyweight from baseline at Day 5 revealed a main effect of sex (*F*(1, 36) = 18.84, *p* = 0.0001), a main effect of diet (*F*(1, 36) = 13.18, *p* = 0.0009), and a significant interaction of diet x sex (*F*(1, 36) = 19.14, *p* < 0.0001). A two-way ANOVA at Day 8 revealed a main effect of sex (*F*(1, 36) = 23.06, *p* < 0.0001), a main effect of diet (*F*(1, 36) = 23.06, *p* < 0.0001), and a significant interaction of diet x sex (*F*(1, 36) = 23.71, *p* < 0.0001). All error bars are S.E.M.

To confirm efficacy of the KD regimen in inducing a ketogenic state, ketone levels were measured in male and female mice that were maintained under ad libitum conditions on either KD or Chow. Ketone levels were measured prior to diet initiation (day 1), and on days 4 and 7 post-diet induction, which revealed a significant increase in β-hydroxybutyrate levels by day 4 for both sexes (Fig. 1A and B). The male mice consistently lost weight on the KD relative to Chow males over the course of eight days (Fig. 1C). In contrast, the female mice displayed no differences in bodyweight between KD and Chow groups over the course of eight days (Fig. 1D).

### Locomotor activity

**Figure 2:**
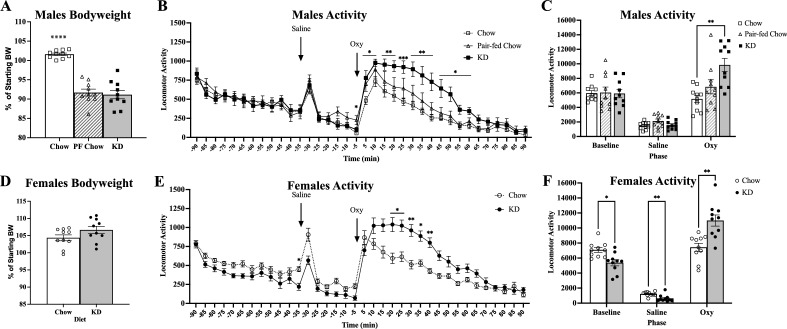
KD increases oxycodone-induced locomotor activity in males and females. (**A**) Changes in bodyweight for the Chow, Pair-fed Chow (PF Chow) and KD groups for the males (n = 10, 10, 10). One-way ANOVA (*F*(2, 27) = 50.45, ****p < 0.0001). (**B)** Male mice two-way ANOVA analysis of binned data revealed a main effect of diet (*F*(2, 27) = 3.585, *p* = 0.0416), a main effect of time (*F*(4.712, 127.2) = 46.48, *p* < 0.0001), and an interaction of diet x time (*F*(70, 945) = 3.122, *p* < 0.0001). A Tukey’s post hoc analysis revealed an increase in activity in KD vs Chow mice (*p ≤ 0.05, **p ≤ 0.01, ***p ≤ 0.001) after oxycodone treatment. Pair-fed Chow mice only showed significant differences from other groups at − 5 min vs Chow, and vs KD at 55 min (*p ≤ 0.05 for both timepoints). (**C)** A two-way ANOVA of cumulative locomotor activity for all three phases of assessment revealed a main effect of diet (*F*(2, 27) = 3.585, *p* = 0.0416), time (*F*(1.437, 38.81) = 90.12, *p* < 0.0001), and an interaction of diet x time (*F*(4, 54) = 6.965, *p* < 0.0001). Tukey’s post hoc analysis revealed an increase in activity in KD vs Chow mice (**p = 0.0014) after oxycodone treatment. (**D**) Changes in bodyweight for females (n = 10,10). (**E**) A two-way ANOVA analysis of binned females revealed a difference in locomotor activity in Chow vs KD groups with a main effect of time (*F*(5.256, 94.60) = 50.21, *p* < 0.0001) and an interaction of diet x time (*F*(35, 630) = 10.47, *p* < 0.0001). Sidak’s post hoc analysis revealed an increase in activity for Chow vs KD mice (-30 min, *p = 0.0318), and an increase in activity for KD vs Chow after oxycodone treatment (*p < 0.05, **p < 0.01). (**F**) Two-way ANOVA analysis of cumulative total locomotor activity for females for each phase revealed a main effect of time (*F*(1.323, 23.81) = 205.5, *p* < 0.0001), no main effect of diet, and an interaction of diet x time (*F*(2, 36) = 22.53, *p* < 0.0001). Sidak’s post hoc analysis showed increased locomotor activity in Chow vs KD females during baseline (*p = 0.0143) and saline (**p = 0.0097), and increased activity by KD females vs Chow in response to oxycodone (**p = 0.0038). All error bars are S.E.M.

To assess general changes in sensitivity to oxycodone due to the KD, we assessed locomotor activity in separate cohorts of male and female mice before and after an acute oxycodone challenge. The male cohort included a Pair-fed Chow group to account for the potential contribution of bodyweight loss on oxycodone sensitivity and response. KD, Chow, and Pair-fed Chow groups were placed on their respective diets for a minimum of four days. β-hydroxybutyrate levels (mM) were assessed in males prior to locomotor testing: Chow (mean = 0.44, StdDev = 0.212), Pair-fed Chow (mean = 0.84, StdDev = 0.135), and KD (mean = 2.42, StdDev = 0.449). A one-way ANOVA revealed a main effect of group (*F*(2,27) = 124.1, *p* < 0.0001). Tukey’s post hoc tests showed that each group was different from the others (Chow vs Pair-fed Chow *p* = 0.015; Chow vs KD and Pair-fed Chow vs KD were both *p* < 0.0001), data not shown. Change in bodyweight percentage from the start of dietary intervention to the time of experiment, showed matched levels between KD and Pair-fed Chow mice, which were both significantly lower than Chow mice (Fig. 2A).

Locomotor activity was recorded during each phase (baseline, saline, and oxycodone). Compared to Chow, KD male mice showed an enhanced locomotor response to oxycodone when measured repeatedly over time as well as the cumulative activity within the oxycodone phase (Fig. 2B, C).

Unlike male mice, female mice showed no differences in the relative change in bodyweight percentage from the start of dietary intervention to the time of locomotor testing (Fig. 2D). Similar to the males, female KD mice displayed an enhanced locomotor response to oxycodone compared to Chow as evaluated across time bins, as well as cumulative activity per phase (Fig. 2E, F). The female Chow mice displayed slightly higher activity than KD mice during the Baseline phase.

### Hot plate

**Figure 3:**
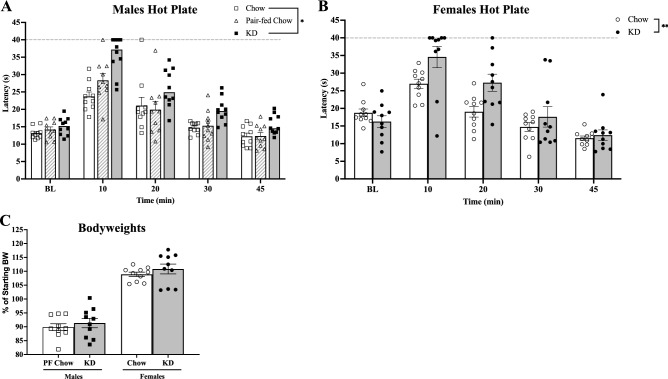
KD increases the antinociceptive effects of oxycodone in the hot plate test in males and females. Males (n = 10,10, 10) on the KD, Pair-fed Chow, or Chow diets, and females (n = 10,10) on the KD and Chow diets were tested at baseline (BL) prior to acute oxycodone, and then at the respective time points. (**A)** Latency to display discomfort of male mice was assessed with a Cox mixed-effects proportional hazards model analysis, revealing a significant main effect of diet (*p = 0.02755), a main effect of time (***p < 0.0001), with a Tukey multiple comparisons test showing an increase in latency in KD vs Chow (*p = 0.024)*.* (**B)** Latency to display discomfort of female mice was assessed with a Cox mixed-effects model analysis of female mice, revealing a significant main effect of diet (**p = 0.0031), a significant main effect of time (*p* < 0.001), and a trend interaction of diet x time (*p* = 0.074; Fig. 3B). **C)** Changes in bodyweight of PF Chow and KD males, as well as Chow and KD females. All error bars are S.E.M.

A hot plate assay was used to assess changes in the antinociceptive effects of oxycodone in males and female mice. Time to nociceptive behavior was recorded at five time points: baseline (BL) prior to 2 mg/kg oxycodone injection, and 10, 20, 30, and 45 min after injection. No difference between male dietary groups was observed at baseline (BL), however latency to show discomfort was significantly increased in the KD mice with a main effect of diet after oxycodone injection (Fig. 3A). Similar to the males, the female mice had no difference at BL between groups, however latency was significantly increased in the KD mice with a main effect of diet after oxycodone (Fig. 3B). There were no differences in bodyweight loss among the Pair-fed Chow vs KD males, and with Chow vs KD females (Fig. 3C).

### Precipitated withdrawal

**Figure 4:**
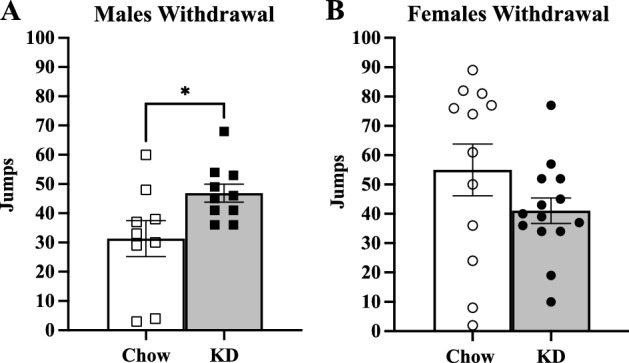
KD results in sex differences in precipitated withdrawal jumps after naloxone treatment. Males (n = 9,10) and females (n = 12,14) that were maintained on the Chow and KD diets respectively were surgically implanted with osmotic minipumps delivering chronic oxycodone over three days. (**A**) Jumping behavior of males after naloxone treatment was assessed by *t*-test (*t* = 2.333, *p = 0.0322). (**B**) Jumping behavior of females after naloxone treatment. All error bars are S.E.M.

To assess the effects of KD on withdrawal symptoms, we measured jumping behavior after inducing withdrawals with naloxone. After mice had completed three days of continuous oxycodone delivery via osmotic minipumps, jumps were quantified over twenty minutes immediately after naloxone injection (1 mg/kg i.p.). Male mice in the KD group had significantly more jumps than Chow mice (Fig. 4A). No difference was observed with female mice comparing KD vs Chow (Fig. 4B).

### Oxycodone self-administration and progressive ratio tests

**Figure 5:**
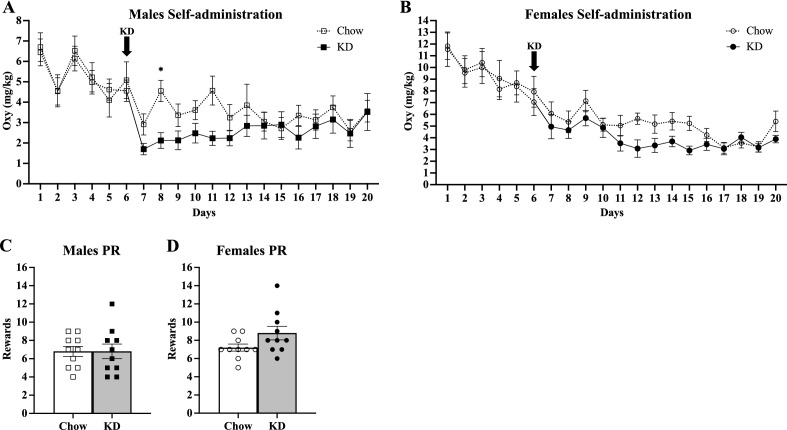
KD reduces oxycodone self-administration in males but has no effect on motivation. All male and female mice (n = 20, 20) were maintained on Chow for training and initial oxycodone self-administration. After their session on Day 6, male (n = 10, 10) and female (n = 10,10) mice were switched to the KD (black arrow) or maintained on Chow and continued their daily self-administration while maintaining food restriction. (**A**) Daily self-administration of oxycodone by males. A two-way ANOVA analysis after diet initiation (session 7–20) showed no main effect of diet or time, but a significant interaction of diet x time (*F* (13, 234) = 2.538, *p* = 0.0028) with KD mice consuming significantly less oxycodone (Sidak’s post hoc analysis *p = 0.0223). (**B**) Daily self-administration of oxycodone by females. Two-way ANOVA revealed a main effect of time (*F* (5.284, 95.11) = 6.689, *p* < 0.0001) and a near significant interaction of diet x time (Fig. 5B; *F* (13, 234) = 1.756, *p* = 0.0510). (**C**, **D**) Progressive ratio testing for males and females. All error bars are S.E.M.

Male and female mice were trained to operantly self-administer oral oxycodone while maintained on standard chow. After their Day 6 self-administration session, the dietary intervention experiment was started by replacing the standard chow with ketogenic diet for the KD group. We observed a significant reduction in oxycodone consumed by KD males (Fig. 5A), and a trend reduction by KD females (Fig. 5B).

These mice were then tested for motivation to acquire oxycodone using a progressive ratio paradigm. Male and female mice showed no difference in rewards earned between KD and Chow groups over the test session (Fig. 5C and D).

## Discussion

These data suggest that KD modulates opioid responses in mice in a sex-specific manner (Table 1). While KD induced substantial weight loss in male mice, this was not the case in female mice despite effective induction of ketosis in both sexes. Strikingly, we found that KD augmented antinociceptive and locomotor effects of oxycodone in both sexes. While KD exacerbated oxycodone withdrawal in males, this effect was absent in female mice. KD also reduced oxycodone self-administration, especially in early sessions following KD diet administration, and with a more pronounced effect in male than female mice. More broadly, these data serve as an example of the importance of nutritional and metabolic state for both therapeutic use of opiates for pain management, as well as treatment of OUD.

**Table 1:** Summary of findings.

Readout	Males: KD versus Chow	Males: PF chow versus Chow	Females: KD versus Chow
Bodyweight			
Locomotor activity (after oxy)			
Antinociception (after oxy)			
Precipitated Jumps (after nalox)		N/A	
Oxy self-administration		N/A	Trend
Oxy rewards earned during PR		N/A	

Up arrows indicate an increase, down arrows indicate a decrease, bidirectional horizontal arrows indicate no change. N/A indicates that group wasn’t included. The effect size (degree of significance) for a given readout is indicated by the number of arrows.

While both male and female mice on KD rapidly develop ketosis, we observed sex differences in bodyweight loss in KD male mice only. This persisted throughout locomotor and hot plate testing. This sex-specific bodyweight response to the ketogenic diet in mice has not been reported in the literature, however it is consistent with clinical data evaluating patients with obesity on a ketogenic diet, where it was reported that male subjects lost more weight than females^29^.

Oxycodone-induced locomotor activity was tested to screen for general changes in opioid sensitivity on the ketogenic diet. Both male and female KD mice displayed robust increases in activity after oxycodone treatment compared to Chow mice, indicating that the ketogenic diet may enhance the effects of the opioid. Interestingly, the female KD mice displayed significantly less activity during baseline and saline phases than Chow. This reversal from lower to higher oxycodone-induced activity strengthens the finding that the dietary manipulation enhanced their opioid sensitivity.

Since it is known that food restriction can enhance behavioral and physiological sensitivity to drugs of abuse^30^, we generated a Pair-fed Chow group matched to the KD group’s bodyweight loss in male mice. β-hydroxybutyrate levels were significantly different across all three male groups prior to locomotor activity testing, which was expected as food restriction can increase ketone levels^31,32^. However, the KD diet produced much higher β-hydroxybutyrate levels than the Pair-fed Chow, and even more importantly, the Pair-fed Chow mice did not show the enhanced locomotor and antinociceptive responses to oxycodone, suggesting that these effects are primarily driven by the ketogenic diet, and not by the bodyweight loss.

We also evaluated effects of the KD on modifying sensitivity to the antinociceptive effects of oxycodone using the hot plate assay. Both male and female KD mice displayed significantly enhanced latency after oxycodone compared to Chow, suggesting that the ketogenic diet may enhance the antinociceptive effects of oxycodone. These data support a potential role for a ketogenic diet as an adjunct with the therapeutic use of opiates for pain management in a clinical setting. In line, as the ketogenic diet has shown efficacy in reducing pain in animals and humans^20,22,33^, it has recently been recommended as a potential adjunct to a comprehensive pain management plan^22^. These data provide more scientific evidence for those recommendations and need clinical validation.

We evaluated KD effects on physical withdrawal symptoms after chronic oxycodone delivery via minipump. In this case, dietary manipulation occurred prior to oxycodone exposure, and withdrawal was evaluated after ketosis had developed. Interestingly, male KD mice had increased jumping behavior after naloxone treatment, suggesting increased sensitivity to withdrawal symptoms, whereas the female KD mice did not show these effects. Again, these data are consistent with an increased sensitivity to oxycodone caused by the KD. These data, along with the sex differences in bodyweight loss, suggest sexual dimorphism of the KD that need further exploration. No studies have investigated directly the influence of sexual dimorphisms of diet on OUD, but several have determined other factors driving OUD responses, such as exercise, weight gain, and also the development of OUD^34–36^.

We also evaluated effects of KD on self-administration of oral oxycodone. After dietary intervention, male KD mice showed a significant reduction in oral self-administration compared to Chow, and female KD mice showed a trend reduction as well. As only one concentration of oxycodone was tested in self-administration and progressive ratio paradigms, future studies evaluating additional concentrations may be of value in confirming translational relevancy. These data may have translational significance by showing that a dietary intervention can impact ongoing opiate consumption. However, clinical studies are necessary to evaluate whether or not this reduction in consumption would have any potential effects on withdrawal symptoms, which in turn might contribute to successful treatment of OUD. Follow up studies are needed to confirm potential efficacy, as well as to test for KD interactions with OUD medications (i.e., methadone, buprenorphine, naltrexone). Notably, neither male nor female KD mice displayed a significant difference in motivation on the progressive ratio schedule, suggesting limited effects of the KD on motivation during intake.

Overall, we show a modulatory role for KD in the response to opioids and opioid withdrawal symptoms. While both sexes experienced improved analgesic effects during opiate use for pain relief, a KD was only efficacious in reducing opiate consumption in male mice, whereas the females exhibited a strong trend in reduction. Meanwhile, withdrawal was selectively enhanced by KD in male, not female, mice. This sex-dependent response profile could have implications for clinical implementation strategies, but direct studies in human subjects are needed. OUD is a complex disorder with a multitude of variables that impact treatment outcomes. Sex differences may be a major factor influencing treatment outcome among OUD patients, with some studies showing that women progress to more severe OUD faster than men, and have higher withdrawal scores, whereas other studies show that men are less likely to remain in treatment^37^. The present findings support the need for an overall awareness and clinical assessment of dietary and metabolic state, as modulated by gender, both during treatment of OUD as well as during the therapeutic use of opiates for pain.

## Data Availability

All data supporting this publication can be available from the corresponding author R.J.D. upon reasonable request.
